# *In silico* Comparison of Left Atrial Ablation Techniques That Target the Anatomical, Structural, and Electrical Substrates of Atrial Fibrillation

**DOI:** 10.3389/fphys.2020.572874

**Published:** 2020-09-16

**Authors:** Caroline H. Roney, Marianne L. Beach, Arihant M. Mehta, Iain Sim, Cesare Corrado, Rokas Bendikas, Jose A. Solis-Lemus, Orod Razeghi, John Whitaker, Louisa O’Neill, Gernot Plank, Edward Vigmond, Steven E. Williams, Mark D. O’Neill, Steven A. Niederer

**Affiliations:** ^1^School of Biomedical Engineering and Imaging Sciences, King’s College London, London, United Kingdom; ^2^Department of Biophysics, Medical University of Graz, Graz, Austria; ^3^IHU Liryc, Electrophysiology and Heart Modeling Institute, Fondation Bordeaux Université, Bordeaux, France

**Keywords:** atrial fibrillation, virtual cohort, catheter ablation, atrial fibrosis, phase singularity mapping

## Abstract

Catheter ablation therapy for persistent atrial fibrillation (AF) typically includes pulmonary vein isolation (PVI) and may include additional ablation lesions that target patient-specific anatomical, electrical, or structural features. Clinical centers employ different ablation strategies, which use imaging data together with electroanatomic mapping data, depending on data availability. The aim of this study was to compare ablation techniques across a virtual cohort of AF patients. We constructed 20 paroxysmal and 30 persistent AF patient-specific left atrial (LA) bilayer models incorporating fibrotic remodeling from late-gadolinium enhancement (LGE) MRI scans. AF was simulated and post-processed using phase mapping to determine electrical driver locations over 15 s. Six different ablation approaches were tested: (i) PVI alone, modeled as wide-area encirclement of the pulmonary veins; PVI together with: (ii) roof and inferior lines to model posterior wall box isolation; (iii) isolating the largest fibrotic area (identified by LGE-MRI); (iv) isolating all fibrotic areas; (v) isolating the largest driver hotspot region [identified as high simulated phase singularity (PS) density]; and (vi) isolating all driver hotspot regions. Ablation efficacy was assessed to predict optimal ablation therapies for individual patients. We subsequently trained a random forest classifier to predict ablation response using (a) imaging metrics alone, (b) imaging and electrical metrics, or (c) imaging, electrical, and ablation lesion metrics. The optimal ablation approach resulting in termination, or if not possible atrial tachycardia (AT), varied among the virtual patient cohort: (i) 20% PVI alone, (ii) 6% box ablation, (iii) 2% largest fibrosis area, (iv) 4% all fibrosis areas, (v) 2% largest driver hotspot, and (vi) 46% all driver hotspots. Around 20% of cases remained in AF for all ablation strategies. The addition of patient-specific and ablation pattern specific lesion metrics to the trained random forest classifier improved predictive capability from an accuracy of 0.73 to 0.83. The trained classifier results demonstrate that the surface areas of pre-ablation driver regions and of fibrotic tissue not isolated by the proposed ablation strategy are both important for predicting ablation outcome. Overall, our study demonstrates the need to select the optimal ablation strategy for each patient. It suggests that both patient-specific fibrosis properties and driver locations are important for planning ablation approaches, and the distribution of lesions is important for predicting an acute response.

## Introduction

Current treatment approaches for persistent atrial fibrillation (AF) are sub-optimal, with over 40% of patients exhibiting AF recurrence within 18 months of catheter ablation therapy (Verma et al., 2015). Catheter ablation typically includes pulmonary vein isolation (PVI) and may include additional lesions. These additional lesions may target patient-specific features of the anatomical, structural, or electrical substrates.

Anatomical ablation approaches aim to either isolate areas that are common sites of triggers or anatomical re-entry (Pambrun et al., 2019), or to reduce the space available for re-entry. Specifically, PVI aims to isolate triggered beats from the pulmonary veins. Linear ablation approaches may include additional ablation lines at the mitral isthmus or the roof of the left atrium; for example, Kottkamp et al. (2002) applied lesion lines from the mitral valve annulus to the pulmonary vein orifices to prevent anatomical reentrant circuits. Yu et al. (2017) demonstrated that linear lesions together with PVI demonstrate similar efficacy to PVI alone. Other anatomical approaches include box isolation, which includes additional ablation lines to isolate a box of tissue on the posterior wall and roof, and aims to reduce the spatial size of the atrial substrate, where fibrillatory wavefronts may persistently propagate (Hwang et al., 2015; Williams et al., 2019). Pambrun et al. (2019) used an ablation strategy that targeted the coronary sinus and vein of Marshall to eliminate potential sites of anatomical re-entry. The anatomical ablation lesion set applied will depend on individual patient anatomy; however, it will not necessarily take into account electrical or structural features of the atrial substrate. We simulate two anatomical ablation approaches: PVI and box isolation.

Structural ablation approaches aim to remove or isolate aberrant fibrotic tissue identified through either electroanatomic mapping or atrial imaging. For example, Kottkamp et al. (2016) performed box isolation of fibrotic areas to remove low voltage areas from electroanatomic voltage maps as a surrogate for fibrotic tissue. The Delayed-Enhancement MRI Determinant of Successful Radiofrequency Catheter Ablation of Atrial Fibrillation (DECAAF) study showed that atrial fibrosis detected on late-gadolinium enhancement MRI (LGE-MRI) was independently associated with AF recurrence (Marrouche et al., 2014; Chubb et al., 2019). The current DECAAFII clinical study aims to investigate whether ablation guided by LGE-MRI is superior to PVI. However, it is challenging to characterize atrial tissue from LGE-MRI imaging data and to use this characterization to decide which areas to ablate. As a further complication, recent studies demonstrate evidence both for and against the colocation of fibrillatory drivers with fibrotic tissue (Haissaguerre et al., 2016; Sohns et al., 2017). We simulate two structural ablation approaches: isolating the largest fibrotic area and isolating all fibrotic areas.

Electrical ablation approaches are personalized to target areas of electrogram fractionation (Nademanee et al., 2004) or to isolate electrical drivers identified using phase singularity (PS) analysis (Lim et al., 2017). Narayan et al. (2012) demonstrated high success rates by ablating focal and re-entrant drivers identified through phase and activation mapping of AF. However, these approaches require accurate mapping of atrial drivers, which is challenging (Handa et al., 2018), and are also complicated by the emergence of post-ablation drivers that may not be present pre-ablation. We simulate two electrical ablation approaches: isolating the largest driver region and isolating all driver regions.

Biophysical modeling provides essential mechanistic insights into the individual contribution of the anatomical, electrical, and structural substrates to AF, and each individual patient’s response to multiple different ablation strategies. This allows the efficacy of specific ablation strategies and the relative effect of ablation strategy and atrial debulking to be predicted. However, biophysical models take a significant amount of time to construct and simulate and so are both compute and resource intensive, limiting their direct clinical applicability. Combining the mechanistic insights of biophysical models with machine learning techniques may lead to a more robust machine learning predictor, which is computationally efficient and allows fast prediction in the catheter laboratory on any desktop computer.

We aimed to compare AF ablation techniques that target features of the anatomical, structural, and electrical AF substrates for patient-specific simulations of paroxysmal and persistent AF. Specifically, we applied different anatomical, structural, and electrical ablation strategies to a cohort of 20 virtual paroxysmal AF patients and 30 virtual persistent AF patients. Subsequently, we trained a machine learning random forest classifier to predict ablation response using (a) imaging metrics alone, (b) imaging and electrical metrics, or (c) imaging, electrical, and ablation lesion metrics.

## Materials and Methods

### Patient Cohort

Computational models were constructed from cardiac MRI data for 20 paroxysmal AF and 30 persistent AF patients treated at St Thomas’ Hospital. Paroxysmal and persistent AFs were defined following HRS/EHRA/ECAS/APHRS/SOLAECE guidelines: paroxysmal AF is AF that terminates spontaneously or with intervention within 7 days; persistent AF is continuous AF that is sustained beyond 7 days (Calkins et al., 2018). MRI data consisted of contrast enhanced magnetic resonance angiogram (CE-MRA) scans, which were used to delineate the left atrial (LA) endocardial wall, together with LGE-MRI data, which were processed for fibrosis tissue distribution. Image acquisition details have been previously published (Sim et al., 2019) and are described in the Supplementary Material. Ethical approval was granted by regional ethics committee (17/LO/0150 and 15/LO/1803).

### Geometry Construction

**Figure 1:**
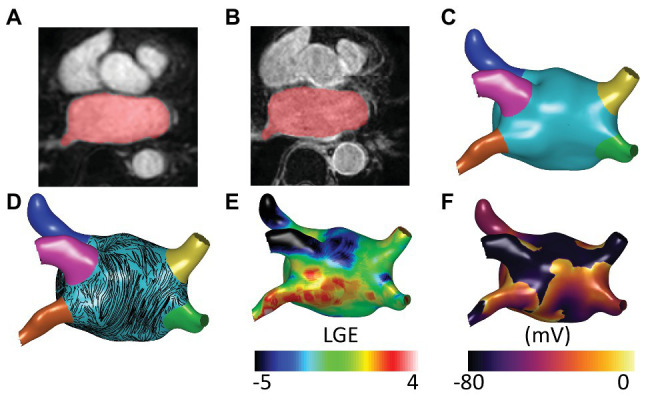
Model construction and atrial fibrillation (AF) simulation. **(A)** Contrast enhanced magnetic resonance angiogram (CE-MRA) imaging showing semi-automatic segmentation of atrial imaging. **(B)** Late-gadolinium enhancement (LGE) imaging with registered segmentation overlaid in red. Anatomical models for each patient were segmented from CE-MRA images and post-processed to create meshes suitable for running biophysical simulations. **(C)** Atrial regions were labeled as follows: left atrial (LA) body (light blue), LA appendage (dark blue), left superior pulmonary vein (pink), left inferior pulmonary vein (orange), right superior pulmonary vein (yellow), and right inferior pulmonary vein (green). **(D)** Atrial fibers from a human atrial *ex-vivo* DTMRI atlas were incorporated using the universal atrial coordinate system, and endocardial fibers are visualized here as streamlines. **(E)** Fibrotic tissue was added through registration of LGE-MRI data. **(F)** AF simulation. AF simulations were run on the models using Cardiac Arrythmia Research package (CARPentry) software. The images included in this figure are an example of one of the virtual patient models of the cohort.

To build an anatomical model for each patient, the left atrium was segmented from the CE-MRA images using semi-automated tools within CemrgApp software^1^ (Sim et al., 2019; Razeghi et al., 2020, see Figure 1A). LGE-MRI scans were registered with CE-MRA images to inform the distribution of fibrotic tissue incorporated into each virtual patient model (see Figure 1B). Each LA segmentation mesh was post-processed using multiple steps, to create a mesh suitable for electrophysiology simulations (See Figure 1C). To create a closed surface, the following filters were applied using Meshlab software^2^: Poisson surface reconstruction, marching cubes, and quadric edge collapse decimation (Cignoni et al., 2008; Kazhdan and Hoppe, 2013). Paraview software (Kitware, Clifton Park, NY, United States^3^; Ahrens et al., 2005) was used to clip the closed surface mesh at the mitral valve and four pulmonary veins, and to label each of the four pulmonary veins and LA appendage (Roney et al., 2019). The clipped and labeled mesh was then re-meshed using mmgtools software^4^ to create triangular elements of approximately equal average edge length (0.34 mm). These steps are shown in Supplementary Figure 1. This endocardial surface mesh was then duplicated and projected 0.1 mm epicardially to produce an epicardial surface, and these were coupled using linear elements to produce a bilayer model (Labarthe et al., 2014). The projection distance is an arbitrary value, since the atrial wall thickness is incorporated in the simulations through the choice of coupling coefficient, following (Labarthe et al., 2014).

Endocardial and epicardial fibers from a human atrial *ex-vivo* diffusion tensor MRI atlas (Pashakhanloo et al., 2016; Roney et al., 2020) were registered to each anatomical mesh using the universal atrial coordinate system (Roney et al., 2019). Specifically, the fiber fields corresponding to diffusion tensor MRI dataset 1 were used because these were shown to optimally predict arrhythmia properties (Roney et al., 2020). Fiber streamlines are shown in Figure 1D. More details on fiber field assignment are given in the Supplementary Material and Supplementary Figure 2. Meshalyzer software^5^ was used to visualize simulation data.

### Biophysical Modeling Details

Biophysical simulations were run using Cardiac Arrhythmia Research Package CARPentry simulator (Vigmond et al., 2003), with the monodomain model for excitation propagation. The Courtemanche–Ramirez–Nattel human atrial ionic model (Courtemanche et al., 1998) was used with the following changes: maximal I_Kr_ conductance was multiplied by 1.6 to represent LA tissue; maximal I_Na_ conductance was multiplied by 2 to ensure physiological action potential upstroke velocities; and maximal I_K1_ conductance was multiplied by 0.8 for a closer agreement with clinical restitution data (Krummen et al., 2012). To incorporate the effects of electrical heterogeneity, the cell model for the LA model was modified as follows for the LA appendage region: maximal I_CaL_ conductance was multiplied by 1.06 and maximal I_to_ conductance was multiplied by 0.67 (Seemann et al., 2006). For the PV region, the following changes were applied to the LA model maximal conductances: g_to_ × 0.75, g_CaL_ × 0.75, g_Kr_ × 1.5, and g_Ks_ × 0.67 (Dorn et al., 2012; Roney et al., 2016). AF electrical remodeling was incorporated in all atrial regions by reducing the maximal ionic conductances of I_to_, I_Kur_, and I_CaL_ by 50, 50, and 70%, respectively, following Courtemanche et al. (1999). Longitudinal conductivity was assigned as 0.4 S/m and transverse as 0.1 S/m.

### Fibrosis Modeling

The effects of fibrotic remodeling were included for each anatomy according to LGE-MRI intensity values (see Figure 1E). Fibrotic remodeling was modeled as regions of conduction slowing together with electrophysiological changes. LGE intensity was normalized for assigning tissue conductivity regions using the maximum and minimum values. Tissue conductivities were then modified to result in 100% conduction velocity in regions of 0–56% normalized LGE intensity; 80% conduction velocity for 56–60% LGE; 60% conduction velocity for 60–64% LGE, and 40% conduction velocity for >64% normalized LGE intensity (Krueger et al., 2014). For electrophysiological remodeling, LGE intensity was rescaled by subtracting the mean blood pool intensity and dividing by the standard deviation (SD). Ionic properties were modified to represent the effects of elevated TGF-ß1 (maximal ionic conductances were rescaled in regions with LGE intensity >3 SDs above the mean of the blood pool as follows: 50% of the regional ionic model value of gK1, 60% of gNa, and 50% of gCaL; Avila et al., 2007; Ramos-Mondragón et al., 2011; Roney et al., 2016; Zahid et al., 2016a). To identify fibrotic regions, LGE-MRI maps were thresholded at 3 SDs above the blood pool mean and separated into different regions using a connected component analysis (Roney et al., 2016).

### AF Induction and Post-processing

Atrial fibrillation was equivalently initiated for each anatomy (see Figure 1F) by setting initial conditions for each simulation which corresponded to four spiral wave re-entries (Matene et al., 2014; Roney et al., 2020). This set-up was defined using the universal atrial coordinate system (Roney et al., 2019) as an activation time field with two Archimedean spirals on each of the posterior and anterior walls, with opposite chirality for adjacent spirals. AF induction is described in more detail in the Supplementary Material and Supplementary Figure 3.

Arrhythmia simulations were post-processed to identify the PS locations for 15 s of arrhythmia data. Spatial PS density maps were calculated using our previous methodology (Roney et al., 2016). To identify regions of high PS density, termed *PS hotspots*, PS density maps were thresholded at 1 SD above the mean and separated into different regions using a connected component analysis.

### Ablation Modeling

We tested six different ablation approaches: (i) PVI alone, modeled as wide-area encirclement of the pulmonary veins; PVI together with: (ii) roof and inferior lines to model posterior wall box isolation (*box* ablation); (iii) isolating the largest fibrotic area (identified as high LGE-MRI intensity; *single fibrosis* ablation); (iv) isolating all fibrotic areas (*all fibrosis* ablation); (v) isolating the largest driver region (identified as high PS density; *single PS hotspot* ablation); and (vi) isolating all driver regions (*all PS hotspots* ablation).

**Figure 2:**
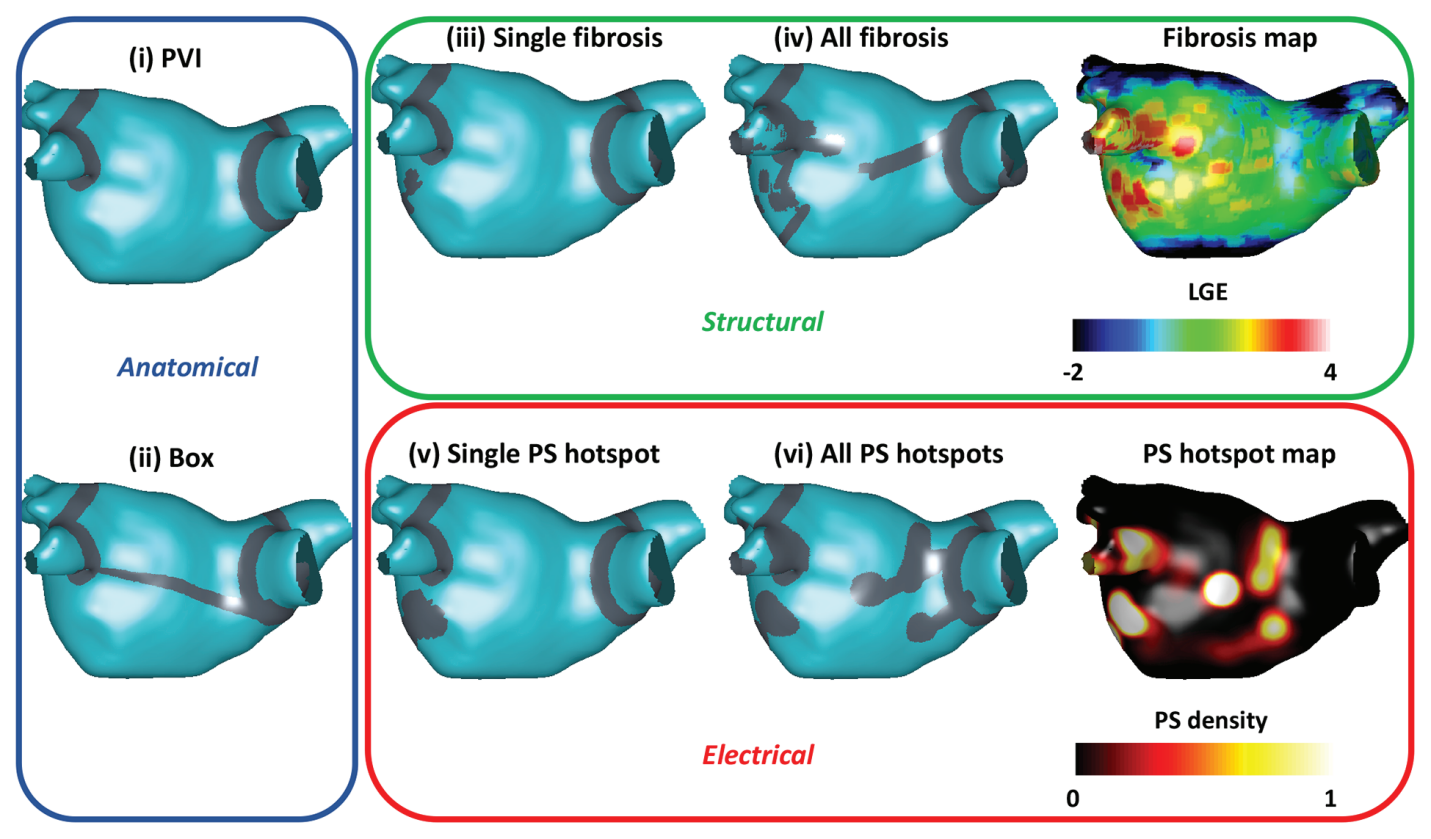
Anatomical, electrical, and structural ablation approaches. The six ablation approaches are indicated by the gray ablation lesions. Anatomical approaches, shown in the blue box, include pulmonary vein isolation (PVI) and box ablation. Structural ablation approaches, shown in the green box, include ablating either a single fibrosis area or all areas of fibrosis on the LGE intensity map. Electrical approaches, shown in the red box, include ablating either a single phase singularity (PS) hotspot or all PS hotspots based on the pre-ablation PS hotspot map.

To automate ablation lesion application and to produce consistent lesions across anatomies, the six different ablation approaches (see Supplementary Figure 4) were defined as follows. PVI was applied at a fixed distance threshold from the junction of the LA body with the pulmonary veins. Roof and inferior lines for the box isolation approach were defined using the universal atrial coordinate system at the same coordinate locations across the virtual cohort (Roney et al., 2019). To identify fibrotic areas for ablation, LGE-MRI maps were first thresholded at 3 SDs above the blood pool mean (Roney et al., 2016). Thresholded tissue was then separated into connected component regions, and the area of each region was calculated. To isolate the largest fibrotic area, the region with the largest area was selected for ablation and joined to the closest mesh boundary or ablation lesion – in this case, either the mitral valve or the PVI lesions. To isolate all fibrotic areas, a lesion set was constructed as PVI together with each fibrotic region joined to either the closest mesh boundary or lesion within the set. Driver region ablation lesions were performed in the same way as fibrosis region ablation but according to PS density maps thresholded at 1 SD above the mean. Figure 2 shows the six ablation approaches for one anatomy in the cohort.

Ablation responses were automatically classified as either termination, macroreentry, or AF. Macroreentry was classified as cases with dominant frequency <4.7 Hz, and AF as cases with dominant frequency >4.7 Hz (Ng et al., 2006; Jarman et al., 2012).

### Random Forest Classifier

Random forest classifiers were trained to predict binary ablation response for three sets of input variables, corresponding to (a) imaging metrics alone; (b) imaging and electrical metrics; and (c) imaging, electrical, and lesion metrics. The imaging metrics used were the total LA body surface area (the surface area of the light blue region in Figure 1A), the total pulmonary vein surface area (the sum of the surface areas of the pink, orange, yellow, and green regions in Figure 1A), and the total fibrotic tissue surface area (thresholded at 3 SDs above the blood pool mean).

The electrical metrics were measured from pre-ablation AF simulations and included the mean dominant frequency and the total PS hotspot area (thresholded at 1 SD above the mean). Five lesion metrics were calculated on the atrial mesh after ablated tissue was removed. Three metrics were calculated in the largest connected region post-ablation as the remaining hotspot area, LA surface area, and fibrosis area (calculated as the remaining tissue area with reduced conductivity values i.e., normalized LGE intensity value >56%). The width of the roof metric was calculated as the width of the largest connected region measured at a universal atrial coordinate value of 0.5 (this is zero in the case of box isolation as the roof is not in the largest connected region post-ablation). The metric corresponding to the smallest channel height post-ablation was calculated as the minimum Euclidean distance between the mitral valve and ablation lesions of significant size (defined as those with area greater than 80% of the second largest ablation lesion).

We split the dataset into training and test sets as a 70:30 split ensuring that all ablation types for a given anatomical model were in the same set. To select hyperparameters for the random forest classifiers, we performed 5-fold cross validation with a balanced-accuracy criterion on the number of estimators, the maximum depth, and the minimum number of samples per leaf. The values tested are given in Table 1. These hyperparameters were then used for the random forest model that was trained using a balanced weighting. Accuracy, precision, and recall were calculated on the test set for the three random forest classifiers. To assess the importance of the input features to the trained random forest classifier, we used the SHapley Additive exPlanations (SHAP) methodology (Štrumbelj and Kononenko, 2014). This was performed using scikit-learn in python, using the functions RandomForestClassifier and GridSearchCV (Varoquaux et al., 2015), and the SHAP toolbox (available at: https://github.com/slundberg/shap).

**Table 1:** Hyperparameter values tested using cross-validation for training random forest classifiers.

Hyperparameter	Values tested
Number of estimators	10, 20, 50, 70, 80, 100
Maximum depth	4, 8, 16
Minimum number of samples per leaf	5, 10, 20

We compared random forest classification to both logistic regression (LogisticRegression in scikit-learn) and support vector machine classifiers (SVCs in scikit-learn), following 5-fold cross-validation to select the optimal hyperparameters.

## Results

### Paroxysmal and Persistent AF Model Ablation Outcomes

Patient characteristics including LA, pulmonary vein and fibrotic tissue surface area calculated from the atrial models are given in Table 2 for each of the paroxysmal and persistent AF cohorts. Supplementary Figure 5 shows that LGE-MRI intensity values and distributions vary across the cohort.

**Table 2:** Patient characteristics calculated from the atrial models for the paroxysmal and persistent AF cohorts.

	Paroxysmal (*n* = 20)	Persistent (*n* = 30)
Left atrial surface area (cm^2^)	102.1 ± 19.0	120.1 ± 23.3
Pulmonary vein surface area (cm^2^)	28.1 ± 7.29	30.4 ± 8.73
Fibrotic tissue surface area (cm^2^)	23.2 ± 7.89	26.2 ± 5.78

The average electrical metrics measured pre-ablation for the cohort were a mean dominant frequency of 4.86 ± 0.11 Hz and a mean total PS hotspot area of 27.7 ± 9.12 cm^2^. Supplementary Figure 6 shows that AF duration varies between models in the cohort. The mean AF duration is 11.0 ± 4.77 s, with 11 cases between 2–5 s, 6 between 5–10 s, 10 between 10–14.9 s, and 23 over 15 s.

**Figure 3:**
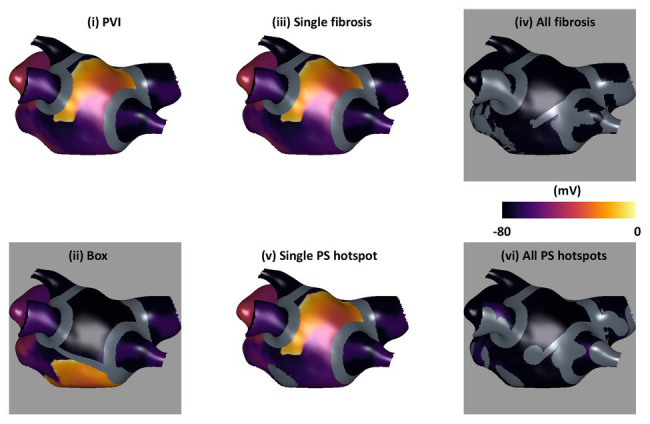
Example transmembrane voltage snapshots demonstrating the six ablation approaches. Plots are shown 1.5 s post-ablation, with ablation lesions overlaid in gray. Ablation response is indicated by background color: responders (AT or termination) are shown with a gray background, and non-responders (AF) with white.

Ablation simulations demonstrated a range of outcomes, which were classified as AF, atrial tachycardia (AT), or termination. Figure 3 shows transmembrane voltage snapshots 1.5 s after each of the six ablation approaches for one virtual patient. For this example, AF continued after three of the ablation strategies (PVI, single PS hotspot ablation, and single fibrosis area ablation); AF converted to AT after box isolation; and the arrhythmia terminated after all PS hotspot ablation and after all fibrosis area ablation. The three AF cases were labeled acute ablation non-responders, and AT and termination cases were labeled acute ablation responders.

Table 3 shows the average lesion metrics for the cohort, calculated as properties of the mesh with ablation lesion sets. These include the following metrics calculated in the largest connected region post-ablation: the remaining hotspot area, LA surface area, and fibrosis area; as well as the width of the roof and the smallest channel height in the post-ablation mesh.

**Table 3:** Average lesion metrics across the virtual cohort for the different ablation approaches.

Property	PVI	Box	Single hotspot	All hotspots	Single fibrosis	All fibrosis
Remaining LA surface area (cm^2^)	90.1 ± 22.4	70.2 ± 19.2	87.1 ± 22.2	80.2 ± 20.6	88.3 ± 21.8	82.9 ± 20.2
Remaining fibrosis area (cm^2^)	32.6 ± 22.9	25.3 ± 19,6	30.1 ± 22.5	25.6 ± 20.5	30.9 ± 23.1	27.1 ± 22.4
Remaining hotspot area (cm^2^)	21.0 ± 8.02	19.6 ± 7.40	15.4 ± 7.08	0.00 ± 0.00	17.9 ± 7.29	13.1 ± 7.05
Conducting roof width (cm)	2.84 ± 1.05	0.00 ± 0.00	2.75 ± 1.14	2.60 ± 1.13	2.83 ± 1.05	2.81 ± 1.06
Smallest post-ablation channel height (cm)	1.96 ± 0.81	1.87 ± 0.79	1.89 ± 0.89	1.02 ± 0.87	1.96 ± 0.82	1.34 ± 0.98

**Figure 4:**
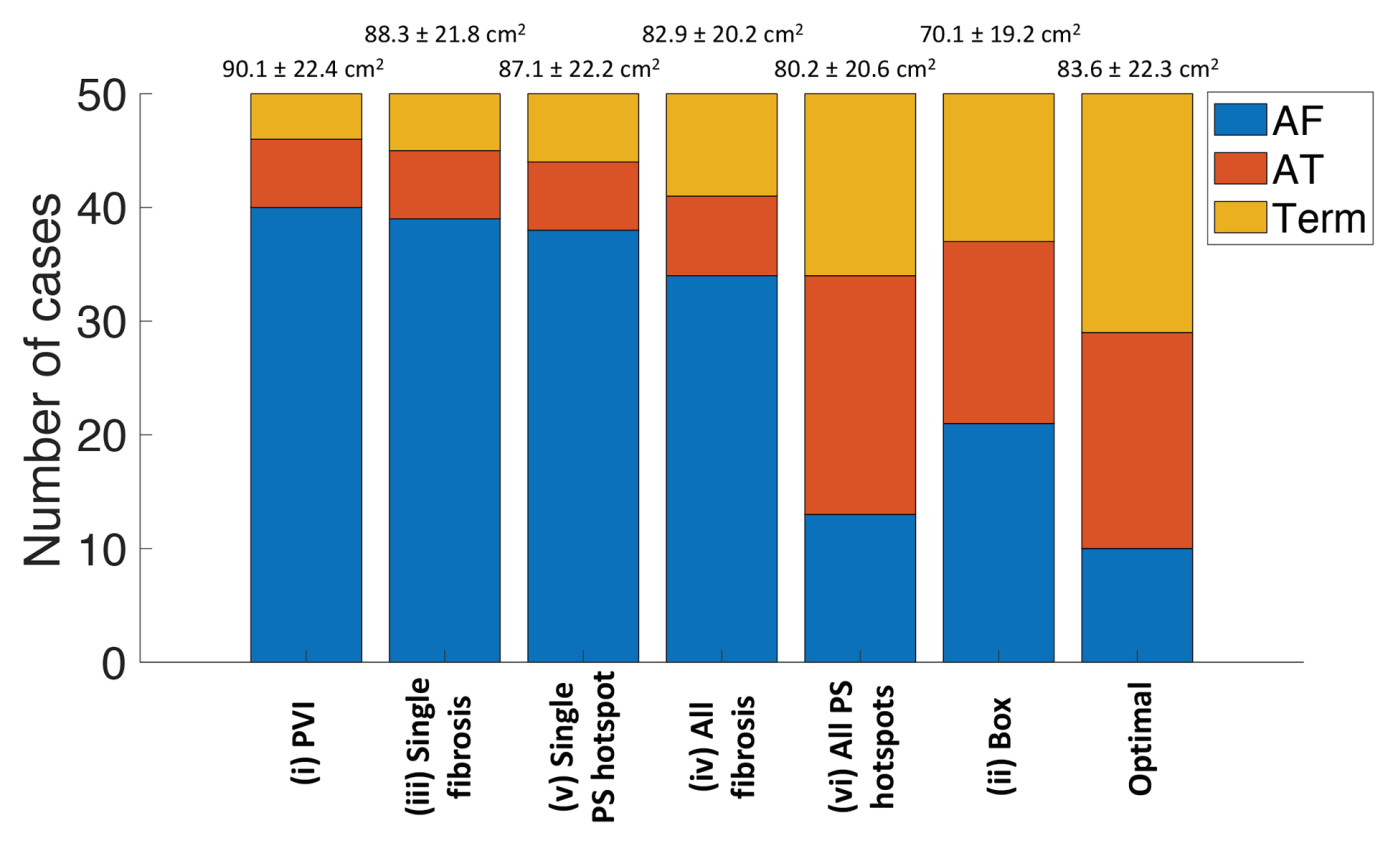
Simulated ablation outcomes classified as AF, AT, or termination varies between the six ablation approaches. The strategies are ordered by the largest area of connected non-ablated tissue in the post-ablation mesh, which are listed as mean and standard deviation (SD) values above the bar chart. The number of outcomes classified as AF is in blue, AT in red, and termination in yellow. The final bar shows the distribution of outcomes if the optimal treatment was chosen for each virtual patient. AF, atrial fibrillation; AT, atrial tachycardia; Term, termination; PVI, pulmonary vein isolation; PS, phase singularity.

Ablation outcomes, classified as AF, AT, or termination, are shown in Figure 4 for the whole virtual paroxysmal and persistent AF cohort with the six ablation strategies. The ablation approaches are ordered by the largest area of connected non-ablated LA tissue in the post-ablation mesh, with PVI resulting in the largest connected area (corresponding to the smallest ablation area), and box resulting in the smallest connected area on average across the cases. This plot shows that ablating all PS hotspots results, on average, in a larger connected area post-ablation than box isolation, but more of the cases converted to AT or terminated. This demonstrates that it is not simply the area of ablated tissue that is important, but also the spatial location of ablation lesions. The final bar of Figure 4 shows the distribution of outcomes if the optimal treatment was chosen for each virtual patient. The optimal treatment was defined as the strategy that results in termination, or if no termination was possible, strategies that resulted in AT were selected. If multiple strategies resulted in the same outcome, the strategy that resulted in the smallest ablation burden was selected. Selecting the optimal treatment for each patient has a greater number of termination and AT cases than any of the six ablation approaches, and results in a larger connected surface area than ablating all PS hotspots.

For 10 of the virtual patients, all ablation strategies tested resulted in AF continuation. These cases are therefore non-responders to the six strategies used. Conversely, for nine of the virtual patients, AF converted to AT or terminated for all six strategies. For each case, we ranked all strategies that result in an acute ablation response by decreasing remaining tissue surface area. Optimal ablation approaches were all driver regions (46%), PVI (20%), box (6%), all fibrosis areas (4%), single driver regions (2%), and single fibrosis area (2%). Twenty percentage of cases remained in AF for all ablation strategies.

Supplementary Table 1 shows that the methodology used for modeling atrial fibrosis and tuning model properties affects the predicted ablation outcome.

### Predicting Outcome Using Random Forest Classifiers

There are many factors that contribute to the continuation of AF. We used a machine learning classifier to identify the factors that predicted response to ablation strategies. To predict ablation response for the virtual patients, we trained random forest classifiers with the following input variables: (a) imaging metrics alone; (b) imaging and electrical metrics; and (c) imaging, electrical, and lesion metrics. Optimal hyperparameters were 50 estimators, a maximum depth of 8, and a minimum number of samples per leaf of 5. The accuracy, precision, and recall measured on the test set were as follows for each of the three classifiers: (a) 0.72, 0.73, and 0.72; (b) 0.73, 0.74, and 0.73; and (c) 0.83, 0.85, and 0.83. As such, the addition of lesion metrics to the model improves its predictive capability compared to simply including the ablation type. The effects of the choice of classifier were tested by also training a logistic regression and SVC. Using all input variables (imaging, electrical, and lesion metrics), the accuracy, precision, and recall on the test set were lower for the trained logistic regression: 0.67, 0.69, and 0.67, and for the trained SVC: 0.76, 0.76, and 0.76.

SHapley Additive exPlanations analysis was performed to determine the relative importance of each variable to the classifier prediction. For classifiers (a) and (b), the type of ablation applied was the most important feature for the prediction. For the imaging metric model, the total fibrosis area and the total LA surface area were the next most important variables, where higher values of each were more likely to be associated with AF post-ablation. For the imaging and electrical metric model, this was also the case, and electrical metrics were less important.

**Figure 5:**
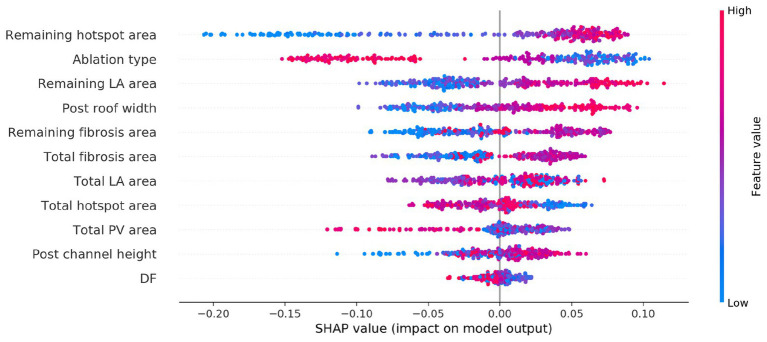
SHapley Additive exPlanations (SHAP) variable importance plot for the trained classifier using imaging, modeling, and ablation lesion metrics. Variables are ranked by the SHAP analysis in descending order of importance, with a red or blue color indicating a high or low value for the observation, respectively.

Figure 5 shows the SHAP summary plot for the combined imaging, electrical, and lesion metrics model. Each point represents a variable value for one of the 300 cases. The horizontal location indicates whether the effect leads to a higher or lower predicted probability (with 0 as responder and 1 as non-responder). Blue points indicate a low value, and red points indicate a high value for an observation. Each of the following lesion metrics was found to be positively correlated with positive prediction (AF, non-responder): remaining hotspot area, remaining fibrosis area, post-ablation roof width, and remaining LA surface area.

### Effects of Lesion Metrics on Acute Ablation Response

**Figure 6:**
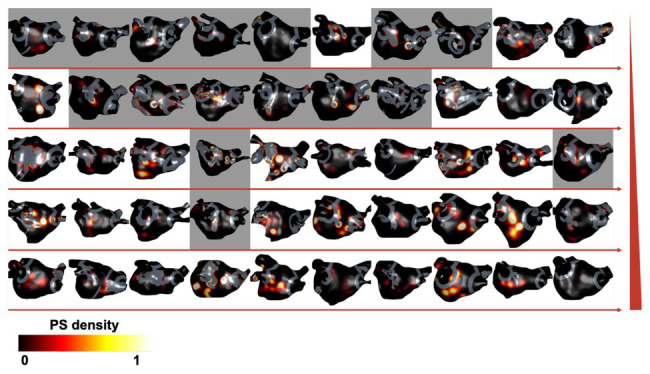
Remaining PS hotspot areas affect ablation outcome. Pre-ablation PS hotspot maps are shown for each virtual patient with ablation lesions targeting all fibrosis regions (ablation strategy iv) overlaid in gray. Cases are ordered by increasing remaining PS hotspot area, shown by directions of horizontal red arrows and increasing by row, indicated by the red vertical ramp. The background color indicates acute ablation outcome: gray for responders (AT or termination) and white for non-responders (AF). There are more acute responders for cases with low than high remaining PS hotspot areas.

The SHAP analysis highlighted that the remaining pre-ablation PS hotspot area following ablation is a key determinant of AF termination. As an example of this, Figure 6 shows PS hotspot maps with ablation lesions that targeted all fibrosis regions indicated in gray on the atrial shell. The maps are shown in order of increasing remaining PS hotspot area, with background color indicating acute ablation outcome (gray for responders and white for non-responders). There are more acute responders for low than high remaining PS hotspot areas. This is indicated by a higher number of maps with a gray background in the upper rows of the figure.

**Figure 7:**
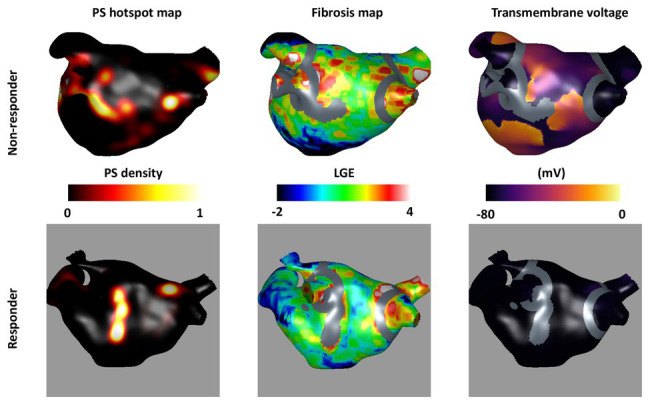
Non-isolated fibrosis area affects ablation outcome. The top row shows an acute non-responder and the bottom row shows an acute responder to the single PS hotspot ablation strategy (strategy number v). For each, the pre-ablation PS hotspot map is shown in the first column; the fibrosis intensity map in the second column; and a transmembrane voltage map at 1.5 s post-ablation in the third column. Ablation lesions corresponding to the largest PS hotspot are shown in gray overlaid on the fibrosis maps and transmembrane voltage maps. There is a larger area of non-ablated fibrosis for the largest post-ablation tissue region in the top row (47.9 cm^2^) than the bottom row (1.57 cm^2^) suggesting that non-isolated fibrosis area affects ablation outcome.

The area of fibrosis remaining post-ablation was also found to be an important factor for predicting ablation outcome. Figure 7 shows an example of cases with high and low remaining fibrosis area following single PS hotspot ablation. The case with a larger non-isolated fibrosis area (47.9 cm^2^) shown in the top row is an acute ablation non-responder since AF continues post-ablation, while a case with a smaller non-isolated fibrosis area (1.57 cm^2^) converts post-single PS hotspot ablation.

## Discussion

### Main Findings

This virtual patient cohort study demonstrates the use of a simulation and a machine learning platform for trialing and analyzing different ablation approaches. We present an efficient pipeline for constructing models from LGE-MRI imaging data (4.5 h from imaging to patient-specific model with DTMRI fibers, regional heterogeneity, and fibrotic remodeling), which we utilized to generate the first cohort of atrial models with fibers from a DTMRI atlas and the largest cohort of atrial models constructed from LGE-MRI data. Specifically, we automatically applied six ablation approaches that target features of the anatomical, structural, or electrical AF substrates to 20 paroxysmal and 30 persistent patient-specific models. Optimal ablation approaches in order of prevalence were all driver regions (46%), PVI (20%), box (6%), all fibrosis areas (4%), single driver regions (2%), and single fibrosis area (2%). Around 20% of cases remained in AF for all ablation strategies. Randomized controlled trials have answered some of the questions regarding ablation of long-standing persistent AF; however, the critical question of technique over debulking persists (Brooks et al., 2010). We show that optimal outcomes require targeting different ablation strategies in different patients and that targeting the AF substrate can be effective beyond its effect on debulking the atria. Overall, our study suggests that both patient-specific fibrosis properties and driver locations are important for planning ablation approaches, and the distribution of lesions is important for predicting an acute response.

### Anatomical Ablation Approaches

We observed a variation in the outcome of the six ablation approaches used across the cohort of models constructed (Figures 3, 4). For example, PVI ablation alone was sufficient for an acute response – and hence the optimal ablation approach – for 20% of the virtual patient models. Lim et al. (2015) reported a higher acute AF termination rate with PVI of 30–60% across multiple clinical trials. This value may be lower in our study because we only studied the ability of the atria to sustain rather than initiate AF; the significant impact of trigger removal on outcome is therefore not captured by our current model. The box ablation lesion set applied in our study converted AF to either AT or termination for 58% of the virtual patient models (Figure 4). However, the box approach removes a large area of LA tissue and so this approach was optimal for only 6% of these models. This success rate could be improved by applying a patient-specific box size and location depending on the individual patient conduction and repolarization properties. Williams et al. (2019) proposed an approach for targeting box ablation lesion sets depending on the patient-specific electrical size. This proposition should also be considered when aiming to achieve a greater success rate.

### Structural Ablation Approaches

Several clinical centers target the fibrotic substrate during ablation therapy to remove or isolate fibrotic tissue which may or may not anchor electrical drivers. Fibrotic tissue can be identified through either electroanatomic mapping (Kottkamp et al., 2016) or atrial imaging (Cochet et al., 2015). In our study, we simulated ablation of fibrotic areas identified from LGE-MRI data. The area of fibrotic tissue in the largest remaining tissue region post-ablation was found to be important for predicting ablation response (Figure 7). This suggests that identifying fibrotic tissue either through imaging or electroanatomic mapping is important, and the choice of measuring modality is likely to affect the identified substrate. However, our findings also indicate that structural ablation approaches targeting fibrotic tissue areas were not as successful as electrical ablation approaches targeting PS driver hotspots. Thus, it may be important to map or predict using simulations, the distribution of electrical drivers when planning ablation therapy and fibrosis imaging alone may not be sufficient to guide ablation in all cases.

### Electrical Ablation Approaches

Ablating all PS driver hotspots was the most effective ablation strategy in our study; resulting in a positive response for 74% of cases and represented the optimal approach for 46% of cases. This suggests that PS hotspots play a key role in driving AF (Figure 7). However, despite ablating all the PS hotspots identified through pre-ablation simulation processing, 26% of cases were still able to sustain AF. This suggests that not all possible PS hotspots are identified during a single AF episode, motivating the methodology of Boyle et al. (2019) that identifies different driver locations through different AF initiation protocols. Narayan et al. (2012) demonstrated high success rates by ablating focal and re-entrant drivers identified through phase and activation mapping of basket catheter data (Schricker and Zaman, 2015). Ensuring the correct classification of phase singularities is critical for targeting ablations because wavefront break up does not represent an equal target to a stable rotor. An optimal method of ablation likely involves an appropriate combination of anatomical, structural, and electrical ablation approaches.

### Comparison With Other Ablation Simulation Studies

There is a variation between both clinical and simulation studies in the ablation approaches utilized. We joined ablation lesions to their closest boundary similar to the study of Weiss et al. (2016) which showed that ablating from the center of a mother rotor to a boundary terminated the arrhythmia. Shim et al. (2017) compared persistent AF ablation using empirically chosen ablation lesion sets (*n* = 55) to simulation guided lesion sets (*n* = 53), chosen from five different lesion sets (PVI, three linear ablations, and one electrogram-guided ablation). They demonstrated that ablation guided by simulations was feasible in clinical practice and not inferior to empirically chosen ablation lesion sets. Bayer et al. (2016) performed a simulation study to compare (1) PVI with roof and mitral lines; (2) circles, lines, or crosses near rotor locations; and (3) 4–8 lines applied to streamline the patient-specific sinus rhythm activation sequence. They found that streamlining activation sequences is a robust alternative ablation approach for cases where other approaches do not terminate AF. This represents a further ablation pattern that could be tested using our simulation and machine learning methodology. In addition, Roney et al. (2018) used a virtual pilot clinical study to predict whether ablating interatrial connections would return the right atrium to sinus rhythm. Our current study could be extended to biatrial meshes to investigate the importance of the right atrium in AF. In a pioneering clinical trial, the optimal target identification *via* modeling of arrhythmogenesis approach clinically ablates fibrotic tissue identified as an ablation target using computational simulations (Boyle et al., 2019).

### Designing Patient-Specific Lesion Sets

We have developed a virtual patient cohort that can be used to predict the optimal ablation strategy for a given patient. Our results (Figure 4) demonstrate that it is not simply the area of ablated tissue that is important in determining ablation outcome, but also the spatial location of ablation lesions in relation to the anatomy, fibrotic tissue distribution and driver positions, and necessitating patient-specific therapy. While providing mechanistic understanding, current simulation strategies take extensive time to create, simulate, and analyze the model output. We have shown that with a limited dataset we can create a classifier with accuracy of 0.83. Increasing the dataset size or number of features may increase the classifier accuracy. Alternately, the simulation-trained classifier could be used to initialize a classifier based on clinical data to accelerate learning from smaller clinical data sets. In addition, the classifier could be used together with a minimal cut analysis to find a successful ablation approach that minimizes ablation area and maximizes the area of conducting tissue (Zahid et al., 2016b).

### Limitations

The results of our simulation predictions need to be compared to the clinical ablation approach and outcome. Our study investigates whether an arrhythmia can be sustained and does not include the effects of triggered beats. There was no significant difference between paroxysmal and persistent virtual patient ablation outcomes in our study. This may be because we did not simulate the effects of triggered beats for initiating AF. We did not include personalized electrophysiology in our simulations, and this may affect AF properties and ablation outcome. Similar to previous studies (Roney et al., 2016), our current study shows that the modeling methodology used for incorporating the effects of atrial fibrosis affects simulation outcome. Future studies should optimize the choice of fibrosis modeling methodology through comparison with clinical outcome. In addition, we did not model the effects of variable wall thickness, which has been shown to affect PS stability and meander (Roy et al., 2018). We performed monodomain simulations, and although the differences with bidomain simulations for paced activation have been shown to be small (Potse et al., 2006), differences for our AF simulations should be investigated. We only included 50 patient-specific models which are insufficient to predict optimal ablation pattern for the six approaches simulated. We simulated and predicted acute outcome, which while correlated with, is not equivalent to long term outcome (Lim et al., 2015). We joined ablated regions to their closest region with additional ablation lines to avoid islands of ablated tissue causing re-entry; however, how best to do this requires further investigation.

## Conclusion

Overall, our virtual cohort study has demonstrated the importance of considering the effect of patient-specific fibrosis properties and driver locations when planning ablation approaches. It is important to consider these factors and the distribution of lesions in order to select the optimal ablation strategy for each patient.

## Data Availability Statement

The raw data supporting the conclusions of this article will be made available by the authors, without undue reservation.

## Ethics Statement

The studies involving human participants were reviewed and approved by regional ethics committee (17/LO/0150 and 15/LO/1803). The patients/participants provided their written informed consent to participate in this study.

## Author Contributions

CR and SN conceived and designed the study. CR, MB, and SN drafted the manuscript. CR, MB, and AM constructed atrial models. CR developed the model construction pipeline, ran the simulations, and analyzed the data. CC and RB helped build the fiber atlas used in this study. OR and JS-L developed CemrgApp segmentation software. EV developed meshalyzer visualization software. EV and GP developed CARPentry simulation software. IS, JW, LO’N, and SW collected and segmented atrial imaging data. SN, SW, and MO’N provided supervision. All authors contributed to the article and approved the submitted version.

### Conflict of Interest

The authors declare that the research was conducted in the absence of any commercial or financial relationships that could be construed as a potential conflict of interest.

## Footnotes

^1^www.cemrgapp.com

^2^www.meshlab.net

^3^www.paraview.org

^4^www.mmgtools.org

^5^https://git.opencarp.org/openCARP/meshalyzer
