# Supplementary material for: In silico Comparison of Left Atrial Ablation Techniques That Target the Anatomical, Structural, and Electrical Substrates of Atrial Fibrillation
Source: Front Physiol. 2020 Sep 16;11:1145. doi: 10.3389/fphys.2020.572874 (PMC7526475; doi:10.3389/fphys.2020.572874)
Supplement: Supplementary file 1 [file Data_Sheet_1.pdf]

Supplementary material for: In silico comparison of left atrial ablation techniques that target the anatomical, structural and electrical substrates of atrial fibrillation

Caroline H Roney[1], Marianne L Beach[1], Arihant Mehta[1], Iain Sim[1], Cesare Corrado[1], Rokas Bendikas[1], Jose Alonso Solis-Lemus[1], Orod Razeghi[1], John Whitaker[1], Louisa O'Neill, [1] Gernot Plank[2], Edward Vigmond[3], Steven E Williams[1], Mark D O'Neill[1], Steven A Niederer[1]

[1] School of Biomedical Engineering and Imaging Sciences, King's College London, London, UK

[2] Department of Biophysics, Medical University of Graz, Graz, Austria

[3] IHU Liryc, Electrophysiology and Heart Modeling Institute, fondation Bordeaux Université, 33600, Pessac, Bordeaux, France

Patient cohort – Image acquisition

Computational models were constructed from cardiac magnetic resonance imaging (MRI) data for 20 paroxysmal AF and 30 persistent AF patients treated at St Thomas' Hospital. Paroxysmal and persistent AF were defined following HRS/EHRA/ECAS/APHRS/SOLAECE guidelines: paroxysmal AF is AF that terminates spontaneously or with intervention within seven days; persistent AF is continuous AF that is sustained beyond seven days (Calkins et al., 2017). Cardiac magnetic resonance imaging was performed on 1.5T MR scanners (Ingenia, Philips Healthcare, Best, Netherlands and Magentom Aera, Siemens Leipzig, Germany). MRI data consisted of contrast enhanced magnetic resonance angiogram (CE-MRA) scans, which were used to delineate the left atrial endocardial wall, together with late gadolinium enhancement (LGE) MRI data, which were processed for fibrosis tissue distribution. CE-MRA data were acquired 90 seconds after gadolinium 0.2 mmol/kg (Gadovist, Bayer HealthCare Pharmaceuticals, Berlin, Germany) administration at a rate of 0.3ml/s. LGE-MRI data were acquired using a 3D, inversion

recovery, spoiled gradient echo acquisition, performed 20 minutes after administration of gadolinium. To minimise the respiratory gating artefact, the respiratory navigator was positioned over the right posterior aspect of the diaphragm as laterally as possible. To ensure adequate nulling of the ventricular myocardium, the inversion time was determined from an inversion time mapping sequence performed immediately prior to LGE-MRI acquisition.

Geometry construction – Delineating atrial regions

Each left atrial segmentation mesh was post-processed using a sequence of steps, to create a mesh suitable for electrophysiology simulations. To create a closed surface, the following filters were applied using Meshlab software (www.meshlab.net): Poisson surface reconstruction, marching cubes, and quadric edge collapse decimation (Kazhdan and Hoppe, 2013), (Cignoni et al., 2008). Following this, Paraview software (Kitware, Clifton Park, NY, USA, www.paraview.org, (Ahrens et al., 2005)) was used to open the closed surface mesh at the mitral valve and four pulmonary veins using the sphere clipping tool; see Figure 1A. Next, each of the four pulmonary veins and left atrial appendage were saved as separate surfaces using the sphere clipping tool in Paraview, see Figure 1B. The clipped mesh shown in Figure 1A was then re-meshed using mmgtools software (www.mmgtools.org) to create triangular elements of approximately equal average edge length (0.34 mm) suitable for biophysical simulations (Roney et al., 2019). Finally, the individual atrial region surface meshes were used to label the atrial regions in the simulation mesh using a custom python script. The final labelled mesh is shown in Figure 1C.

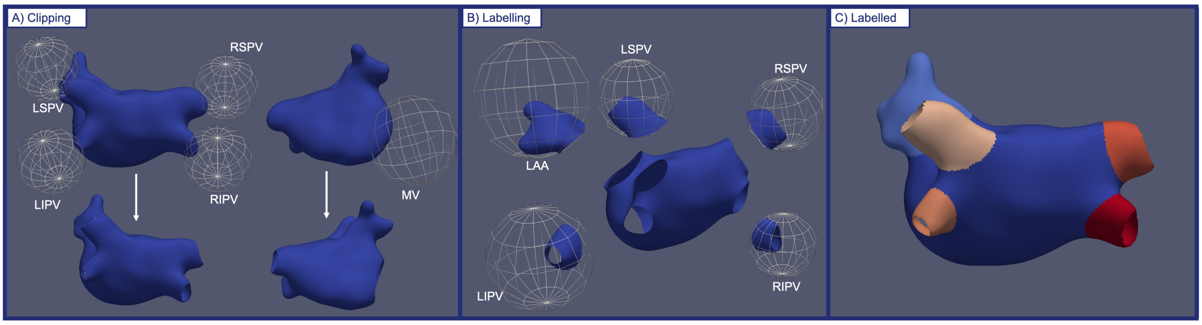

Figure 1: Methodology for labelling atrial regions. (A) Paraview software was used to open each of the four PV and mitral valve annulus using the sphere clipping tool. (B) Each of

the four PV and left atrial appendage were clipped in turn and saved as separate meshes. (C) A custom python script was used to label the individual atrial regions in the simulation grade mesh using the separate meshes. The regions are as follows: LA body (dark blue), LAA (light blue), PV from light pink to dark red (LSPV, LIPV, RSPV, RIPV). Abbreviations are: LSPV (left superior pulmonary vein), LIPV (left inferior pulmonary vein), RIPV (right inferior pulmonary vein), RSPV (right superior pulmonary vein), LAA (left atrial appendage), MV (mitral valve).

The endocardial surface mesh was then duplicated and projected 0.1mm epicardially to produce an epicardial surface, and these were coupled using linear elements to produce a bilayer model (Labarthe et al., 2014). The projection distance is an arbitrary value since the atrial wall thickness is incorporated in the simulations through the choice of coupling coefficient, following (Labarthe et al., 2014). Changes in coupling between the endocardial and epicardial surfaces due to the presence of interstitial fibrosis or varying wall thickness can be incorporated in the model by modifying this coefficient.

Geometry construction – Atrial Fibres

Endocardial and epicardial fibres from a human atrial ex-vivo diffusion tensor MRI atlas (Pashakhanloo et al., 2016) (Roney et al., 2020) were registered to each anatomical mesh using the Universal Atrial Coordinate system (Roney et al., 2019). Specifically, the fibre fields corresponding to diffusion tensor MRI dataset 1 were used because these were shown to optimally predict arrhythmia properties in our previous study (Roney et al., 2020). An example of the endocardial and epicardial fibre fields visualised as streamlines using our previous methodology (Roney et al., 2020) for one virtual patient model is shown in Figure 2.

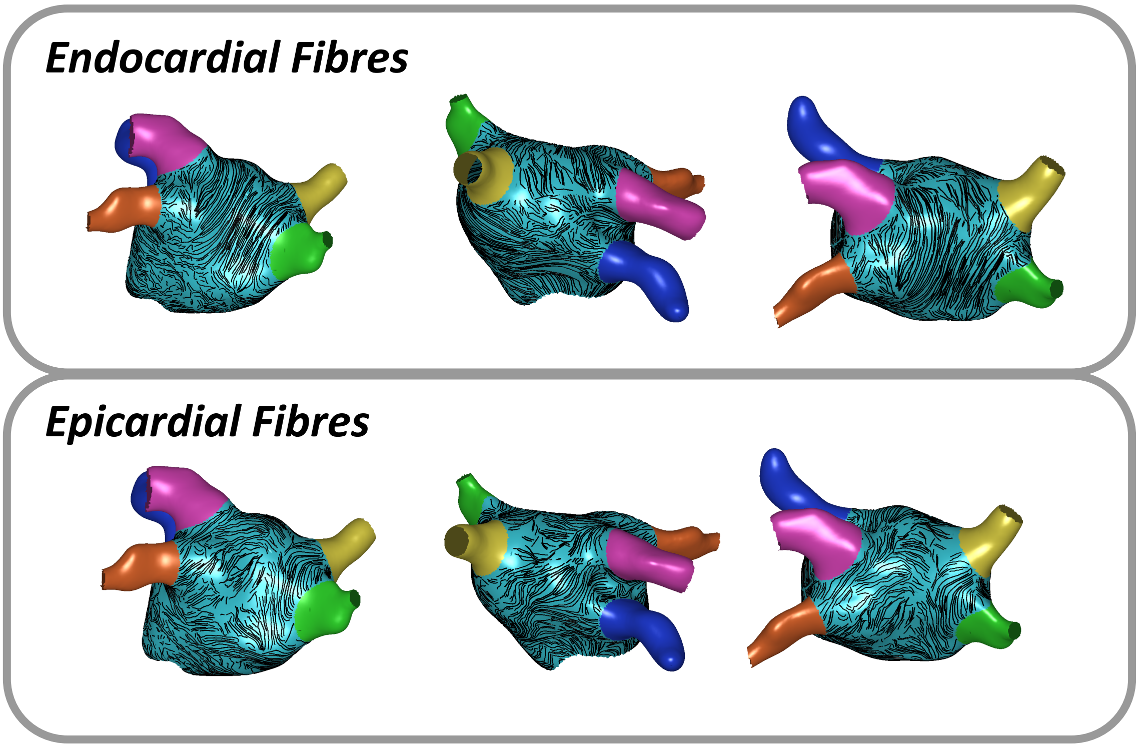

Figure 2: Endocardial and epicardial fibre incorporation. Endocardial and epicardial fibres from a human atrial ex-vivo DTMRI atlas were incorporated in each anatomical mesh using the Universal Atrial Coordinate System. The images included in this figure are an example of one of the virtual patient models of the cohort and show the posteroanterior, anteroposterior and roof views respectively.

AF induction protocol

AF was equivalently initiated for each anatomy by setting initial conditions for each simulation that corresponded to four spiral wave re-entries (Matene et al., 2014) (Roney et al., 2020).

Activation patterns were defined using in built CARP functionality in 4 steps. First, a cell model was paced to estimate the cellular state variables at a given pacing rate (6.06Hz). Second, an analytic expression for activation time was defined for four spiral waves in universal atrial coordinates. Third, the activation times were mapped to specific atrial anatomies, using the universal atrial coordinates. Forth, using the offset activation time, the corresponding cell model membrane potential and state variables were mapped to every location in each patient's mesh. While not accounting for the specifics of the tissue stimulus

or effects of wave curvature, this activation process initialises the tissue with cells in a rapid activation state with an estimate of the offset of cell properties. This methodology is a computationally efficient way to induce reentry. This opencarp tutorial explains the functionality: https://opencarp.org/documentation/examples/02_ep_tissue/03b_study_prep_init.

Figure 3 demonstrates the methodology for an example atrial mesh in the cohort. The atrial mesh was first mapped to 2D universal atrial coordinates (Roney et al., 2019) (Figure 3A). A local activation time field was defined in the unit square universal atrial coordinates system as four Archimedean spirals (Rappel and Narayan, 2013), shown in Figure 3B, with two spirals on each of the posterior and anterior walls, with opposite chirality for adjacent spirals. This local activation time field was then mapped back to the 3D anatomical shell and used as input for the tissue model initialisation. Figure 3C shows this local activation time field as well as the transmembrane potential maps generated by CARPentry using the pre-pacing utility. Simulations are run from this state and AF is sustained (Figure 3D).

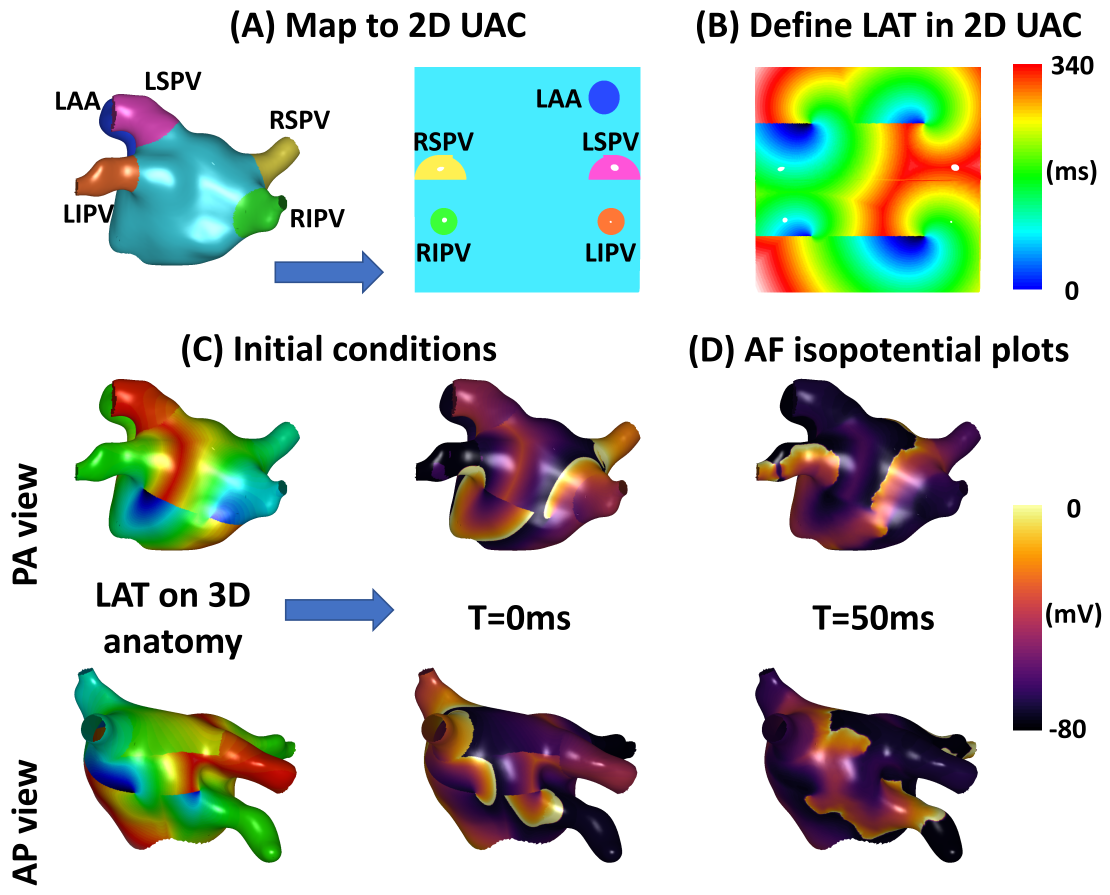

Figure 3: AF induction methodology. (A) The atrial mesh was mapped to 2D universal atrial coordinates (UAC). (B) A local activation time (LAT) field was defined as four Archimedean spirals in 2D UAC. (C) The LAT field was expressed back on the atrial mesh and used as input to generate initial conditions for the voltage distribution, shown in the second column. (D) Isopotential plots showing AF was sustained 50ms post AF induction. For (C) and (D) the top row shows a posteroanterior (PA) view, and the bottom row shows an anteroposterior (AP) view.

Ablation modelling methodology

Figure 4 shows a schematic of the ablation modelling methodology.

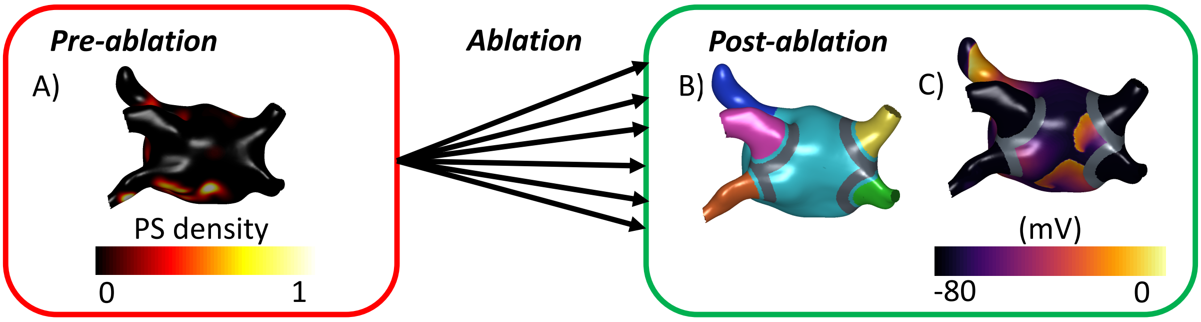

Figure 4: Ablation schematic. (A) AF simulations were run and post-processed to generate PS maps. (B) The models were ablated using one of the six ablation approaches (black arrows). (C) Transmembrane potential map 1.5s post-ablation. The images included in this figure are an example of ablation for one of the virtual patient models of the cohort. In this example a PVI ablation approach was used.

LGE-MRI intensity distributions

Figure 5 shows that LGE-MRI intensity values and distributions vary across the cohort.

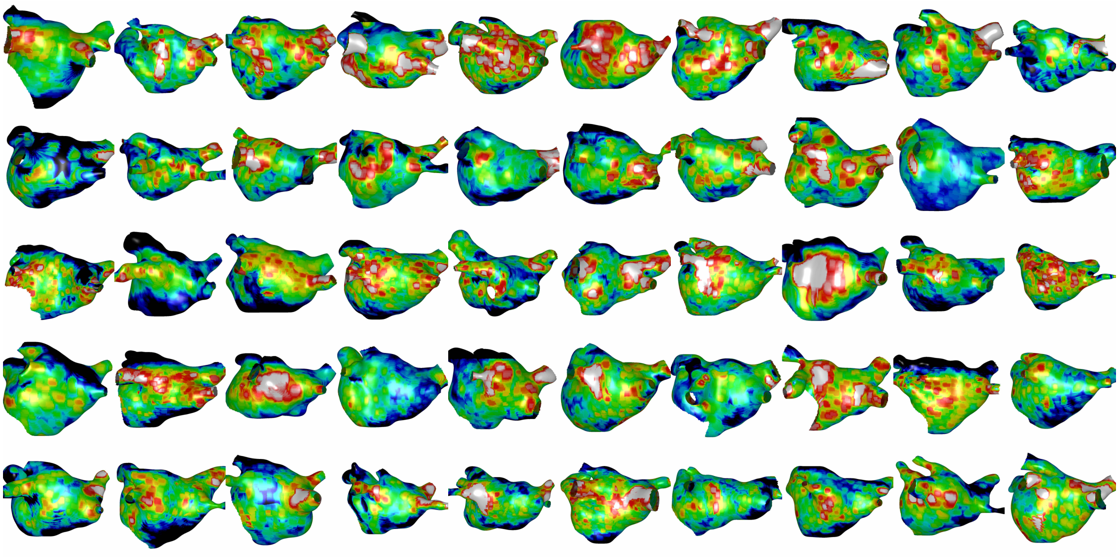

Figure 5: LGE-MRI intensity values and distributions vary across the cohort. The top two rows are 20 paroxysmal patients; the bottom three rows are 30 persistent patients in the cohort.

AF duration pre-ablation

Figure 6 shows AF duration in ascending order across the cohort, demonstrating a range of AF durations pre-ablation. The mean AF duration is 11.0±4.77s, with 11 cases between 2-5s, 6 between 5-10s, 10 between 10-14.9s and 23 over 15s.

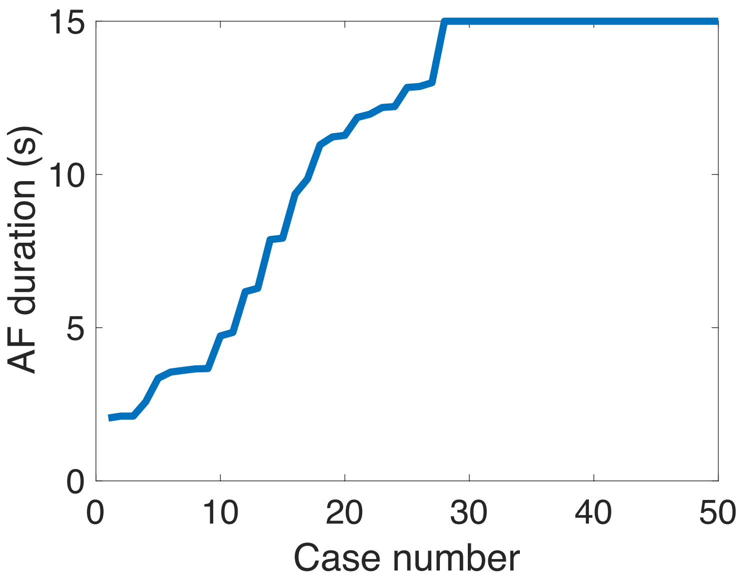

Figure 6: AF duration varies across the cohort.

Effects of fibrosis modelling methodology

To test the effects of the fibrosis modelling methodology on the predicted ablation response, we simulated PVI ablation for models without any fibrotic remodelling (no fibrosis), for models with conductivity changes only (conductivity) and for cases with ionic changes only (ionic), and compared these to the baseline model in this study which had both conductivity and ionic remodelling in fibrotic regions. Table 1 gives the total number of cases classified as AF, atrial tachycardia and termination 2s post PVI ablation for each of the model set-ups. It shows that the methodology used for modelling atrial fibrosis and tuning model properties affects the predicted ablation outcome.

	AF	*Atrial tachycardia*	*Termination*
Fibrosis	40	6	4
No fibrosis	36	4	10
Conductivity	46	2	2
Ionic	33	3	14

Table 1: Model outcome depends on atrial fibrosis modelling methodology. Simulations are classified 2s post PVI ablation as AF, atrial tachycardia or termination.

References:

Ahrens, J., Geveci, B., and Law, C. (2005). "ParaView: An end-user tool for large-data visualization," in *Visualization Handbook* doi:10.1016/B978-012387582-2/50038-1.

Calkins, H., Hindricks, G., Cappato, R., Kim, Y. H., Saad, E. B., Aguinaga, L., et al. (2017). 2017 HRS/EHRA/ECAS/APHRS/SOLAECE expert consensus statement on catheter and surgical ablation of atrial fibrillation. *Europace* 20, e1–e160. doi:10.1093/europace/eux274.

Cignoni, P., Callieri, M., Corsini, M., Dellepiane, M., Ganovelli, F., and Ranzuglia, G. (2008). MeshLab: An open-source mesh processing tool. in *6th Eurographics Italian Chapter Conference 2008 - Proceedings*.

Kazhdan, M., and Hoppe, H. (2013). Screened poisson surface reconstruction. *ACM Trans. Graph.* doi:10.1145/2487228.2487237.

Labarthe, S., Bayer, J., Coudière, Y., Henry, J., Cochet, H., Jaïs, P., et al. (2014). A bilayer

model of human atria: mathematical background, construction, and assessment. *Europace* 16 Suppl 4, iv21–iv29. doi:10.1093/europace/euu256.

Matene, E., Vinet, A., and Jacquemet, V. (2014). Dynamics of atrial arrhythmias modulated by time-dependent acetylcholine concentration: a simulation study. *Europace* 16 Suppl 4, iv11–iv20. doi:10.1093/europace/euu255.

Pashakhanloo, F., Herzka, D. A., Ashikaga, H., Mori, S., Gai, N., Bluemke, D. A., et al. (2016). Myofiber architecture of the human atria as revealed by submillimeter diffusion tensor imaging. *Circ. Arrhythmia Electrophysiol.* 9, 1–9. doi:10.1161/CIRCEP.116.004133.

Rappel, W.-J., and Narayan, S. M. (2013). Theoretical considerations for mapping activation in human cardiac fibrillation. *Chaos* 23, 023113. doi:10.1063/1.4807098.

Roney, C. H., Bendikas, R., Pashakhanloo, F., Corrado, C., Vigmond, E. J., McVeigh, E. R., et al. (2020). Constructing a Human Atrial Fibre Atlas. *Ann. Biomed. Eng.* doi:10.1007/s10439-020-02525-w.

Roney, C. H., Pashaei, A., Meo, M., Dubois, R., Boyle, P. M., Trayanova, N. A., et al. (2019). Universal atrial coordinates applied to visualisation, registration and construction of patient specific meshes. *Med. Image Anal.* 55, 65–75. doi:10.1016/j.media.2019.04.004.

Vigmond, E. J., Hughes, M., Plank, G., and Leon, L. J. (2003). Computational tools for modeling electrical activity in cardiac tissue. *J. Electrocardiol.* 36, 69–74. doi:10.1016/j.jelectrocard.2003.09.017.
